# Replacing the Classics? A Comparison of the ERPs Evoked by IAPS and OASIS Images During Emotional Processing

**DOI:** 10.1111/psyp.70231

**Published:** 2026-01-20

**Authors:** Valentina Mologni, Carola Dell'Acqua, Simone Messerotti Benvenuti

**Affiliations:** ^1^ Department of General Psychology University of Padua Padua Italy; ^2^ Padova Neuroscience Center University of Padua Padua Italy

**Keywords:** affective processing, ERPs, IAPS, OASIS, S1‐S2 task

## Abstract

Emotional visual stimuli presented in laboratory settings reliably elicit prototypical patterns of subjective and psychophysiological responses. These responses likely serve distinct functions and reflect the engagement of appetitive and defensive motivational systems, making them a valuable tool for examining emotional processing in both healthy individuals and those with mental disorders. Event‐related potentials (ERPs), such as the Cue‐P300, Stimulus Preceding Negativity (SPN), and Late Positive Potential (LPP), provide valuable temporal insight into anticipatory and elaborative stages of emotional processing. While these components have been extensively studied using the International Affective Picture System (IAPS), concerns about its dated content have prompted the development of alternative image sets, such as the Open Affective Standardized Image Set (OASIS). Yet, ERP responses to OASIS images remain underexplored. This study aimed to compare psychophysiological and subjective responses elicited by images from the IAPS and OASIS databases, matched for valence and arousal. Twenty‐three participants completed two emotional S1–S2 tasks—one using IAPS images and the other using OASIS images—while undergoing EEG recording. In each task, a cue (S1) predicted the valence (pleasant, neutral, unpleasant) of an upcoming emotional image (S2). The SPN, Cue‐P300, and the LPP components were analyzed. Results revealed that emotional (pleasant, unpleasant) OASIS images elicited larger Cue‐P300 amplitudes than IAPS emotional images, whereas no SPN component was observed for either database. In addition, both IAPS and OASIS images elicited a robust LPP modulation, with this effect more pronounced for IAPS than OASIS unpleasant images. Together, these results indicate that both IAPS and OASIS images elicit the expected emotional responses. Self‐reported ratings were similar across the two databases, alongside the LPP modulation by emotional content. Although further research is needed to clarify how image selection may influence emotional anticipation, the integration of the two databases appears to be a future viable approach.

## Impact Statement

This study provides novel insights about the role of stimulus selection in shaping neural and subjective responses during different emotional processing stages. By directly comparing event‐related potentials elicited by two standardized image sets—the International Affective Picture System (IAPS) and the more recent Open Affective Standardized Image Set (OASIS)—the findings indicate that both image sets elicit the expected emotional responses at the elaboration stage, suggesting that the integration of the two databases might be a future approach in studies employing emotional pictures. However, further research is needed to clarify whether they also elicit similar anticipatory processes.

## 1 Introduction

Decades ago, researchers began to demonstrate that emotional visual stimuli presented in laboratory settings elicit a prototypic pattern of central and peripheral psychophysiological responses, which likely serve different functions (e.g., attention and mobilization for action) and reflect the engagement of appetitive and defensive motivational systems (Bradley et al. 2001; Codispoti et al. 2001, 2006; Cuthbert et al. 1998). In this context, studies examining responses in both healthy individuals and those with mental disorders have identified different psychophysiological patterns associated with specific conditions (e.g., Bylsma et al. 2008; Wauthia and Rossignol 2016).

Building on this, extensive research has explored how individuals respond to stimuli that vary in hedonic valence (e.g., pleasant, neutral, unpleasant) and arousal (e.g., intensity of motivational activation) across subjective, behavioral, and physiological domains (e.g., Grühn and Scheibe 2008; Lane et al. 1999). Within the latter, event‐related potentials (ERPs) from the electroencephalographic signal are particularly well‐suited to study the processing of emotional stimuli (Luck 2014), as they allow the investigation of functionally distinct yet temporally close processes. Specifically, ERPs allow us to examine two key stages of emotional processing: *emotional anticipation*, which involves the preparatory allocation of attentional and motivational resources to upcoming emotional stimuli (Pastor et al. 2014; Poli et al. 2007); and *emotional elaboration*, involving the orientation of attention and sustained processing of the stimulus upon its presentation (Migita et al. 2011; Schupp et al. 2000).

Research using ERPs to measure emotional anticipation has predominantly relied on two distinct components: the Cue‐P300 and the Stimulus Preceding Negativity (SPN). The Cue‐P300 is a positive deflection that primarily emerges at parietal sites following the presentation of a cue that signals an upcoming emotional stimulus. This component reflects preparatory attentional and affective engagement and is typically larger for emotional stimuli (i.e., pleasant and unpleasant) compared to neutral ones (Novak and Foti 2015), indicating heightened anticipation of emotionally salient events. In addition, the SPN is a slow negative potential that emerges approximately 200 ms before the onset of the anticipated stimulus (S2), with greater amplitude (i.e., increased negativity) observed during the anticipation of emotional compared to neutral stimuli at frontocentral sites (Böcker et al. 1994; Takeuchi et al. 2005). The SPN has been linked to the anticipation of emotionally arousing content (Brunia et al. 2011) and is modulated by the level of motivational engagement with the forthcoming stimulus.

The elaboration of emotional stimuli (e.g., scenes, faces, words) reliably elicits the Late Positive Potential (LPP), a sustained centro‐parietal component which is enhanced when pictures depict emotional, compared to neutral, content (Bradley et al. 2007; Codispoti et al. 2001; Palomba et al. 1997; Schupp et al. 2000). The LPP occurs about 400 ms following stimulus onset and is thought to reflect a heightened intake and processing of motivationally relevant stimuli that facilitate the selection of appropriate actions to maximize survival (Schupp et al. 2000). Notably, the LPP is larger for both highly arousing pleasant and unpleasant stimuli, suggesting that stimulus significance is the key dimension that modulates the amplitude of this component (Hajcak and Foti 2020). In addition, the LPP is not associated with low‐level perceptual properties (e.g., picture size, complexity, color) and it remains stable even when the same image is viewed repeatedly (Ferrari et al. 2017) and across multiple sessions within the same individual (Weinberg et al. 2021). Furthermore, the affective modulation of the LPP has been consistently observed across various tasks, including free viewing, affective categorization, and when emotional images are used as distractors (e.g., Mastria et al. 2017; Micucci et al. 2020). The LPP has attracted considerable attention for its reliability as an index of motivated attention to emotional stimuli and is increasingly used in research examining individual differences in personality and psychopathology (e.g., Dell'Acqua et al. 2022; Grunewald et al. 2019; Hill et al. 2019; Moretta et al. 2021; Speed et al. 2015; Vallet et al. 2020). Indeed, recent findings suggest that the LPP may serve as a valuable marker for both the presence of vulnerability to psychopathology (e.g., Bylsma et al. 2022; Grunewald et al. 2019; Moretta et al. 2021; Moretta and Messerotti Benvenuti 2023; Pedersen and Larson 2016; Vallet et al. 2020).

Many of the studies referenced above relied on images from the International Affective Picture System (IAPS; Lang and Bradley 2007), a widely adopted and publicly available database of emotional pictures developed by the Center for the Study of Emotion and Attention. The IAPS comprises 1195 images, each rated for valence, arousal, and dominance by a large sample of participants (100 ratings per image), and cross‐validation studies have demonstrated that these images consistently elicit reliable subjective and physiological responses (Greenwald et al. 1989; Lang et al. 1993).

Despite its extensive use in affective science, several factors have underscored the need to complement the use of IAPS with alternative databases of emotional images. First, to ensure enough trials, some tasks require the presentation of multiple images for each emotional and semantic category, making it necessary to integrate IAPS with additional image sets. Second, IAPS may pose cultural challenges, as many feature elements (e.g., vehicles, furniture) might be perceived as dated and reflective of past decades. Additionally, the scenes depicted often represent the culture of the United States, which may not be equally relevant or resonant in other countries, potentially leading to idiosyncratic contextual issues. Third, although the LPP remains relatively stable across repetitions (e.g., Codispoti et al. 2006; Weinberg et al. 2021), it is less clear how repeated exposure to the same stimuli might affect other ERPs indexing earlier stages of emotional processing. For example, increased familiarity with images used in laboratory settings may attenuate anticipatory responses as upcoming stimuli become more predictable (Morís et al. 2013; Foti and Hajcak 2012). Therefore, the repeated and exclusive use of the IAPS across multiple tasks or studies may potentially introduce systematic effects related to stimulus familiarity and predictability.

These limitations have motivated the development of alternative and open‐access databases of affective pictures, such as the Open Affective Standardized Image Set (OASIS) developed at Harvard University (Kurdi et al. 2017). The OASIS includes 900 digitalized pictures and provides normative ratings of valence and arousal collected from a sample of 822 participants. However, its application in psychophysiological research remains limited (Engelen et al. 2023; Fiorini et al. 2024; Palatinus et al. 2022). Moreover, many of these studies have only used OASIS images along with pictures from the IAPS or other databases (Dickey, Pegg, and Kujawa 2021; Dickey, West, et al. 2021; Engelen et al. 2023), and a direct comparison of the responses elicited by the OASIS and IAPS images has yet to be conducted.

To address the need for integrating the IAPS with additional image sets that elicit comparable emotional responses and expand the limited psychophysiological research on the OASIS database, this study examined emotional responses elicited by images from both sources, ensuring comparability in terms of valence and arousal based on both psychophysiological measures and subjective ratings. To our knowledge, only two studies have used the OASIS database to measure the LPP (Dickey, Pegg, and Kujawa 2021; Dickey, West, et al. 2021), while no studies so far have employed it to examine the Cue‐P300 and the SPN. Since the OASIS database underwent a validation process similar to that of the IAPS, it was hypothesized that subjective and physiological responses would be comparable across the two databases. Specifically, it was expected larger ERP amplitudes for emotional vs. neutral images during both emotional anticipation (indexed by the Cue‐P300 and the SPN) and emotional elaboration (indexed by the LPP). Additionally, greater self‐reported arousal in response to pleasant and unpleasant – compared to neutral – stimuli was expected in both databases. Similarly, in both databases, lower valence ratings were expected for unpleasant compared to pleasant and neutral stimuli, as well as for neutral compared to pleasant stimuli.

## 2 Methods

### 2.1 Participants

Twenty‐three Italian Caucasian students (15 females, mean (*M*) age = 22.39 years, standard deviation (SD) = 1.89 years, range = 20–26 years) from the University of Padua (Italy) voluntarily participated in the study. In line with the guidelines provided by Gibney and colleagues, a sample size of 23 participants and 28 trials per category is adequate to detect the expected LPP differences in response to high‐ versus low‐arousing images within a simple within‐subject design (Gibney et al. 2020). The enrolled sample was medically healthy and free from psychotropic medication, as assessed with an ad‐hoc interview. Exclusion criteria included a current diagnosis or history of cardiovascular and neurological diseases and a formal diagnosis of mental diseases. As this study was part of a larger project investigating affective processing in individuals vulnerable to depression, only participants without depressive symptoms, as evaluated with the Beck Depression Inventory‐II (BDI‐II, scores < 12; Beck et al. 1996; Sica and Ghisi 2007), were included. All participants had a normal‐to‐corrected vision and were naïve to the purpose of the experiment. All participants understood and signed the informed consent forms. The study adhered to the Declaration of Helsinki on research on human subjects and was approved by the Ethical Committee of Psychological Research, Area 17, University of Padua (prot. no. 564‐b). Participants received a monetary compensation of 25 euros for their participation.

### 2.2 Experimental Task

In this study, participants engaged in an S1‐S2 emotional paradigm (e.g., Poli et al. 2007) while undergoing electroencephalographic (EEG) recording. This task involves the presentation of an emotional image (S2), which is preceded by a cue (S1) anticipating its valence, allowing to explore both the anticipation and the elaboration of emotional stimuli. The task consisted of two blocks of 84 trials each. The two blocks presented images from the two different databases, namely IAPS and OASIS, and the order of the blocks was counterbalanced across participants. Each trial started with a 500 ms baseline (a white fixation dot), followed by a cue (S1) lasting 1500 ms that signaled the emotional content (a plus for pleasant, a circle for neutral, a minus for unpleasant) of the upcoming picture (S2), presented after the 4500 ms inter‐stimulus interval (ISI) and lasting 6000 ms. The S2 was followed by a variable inter‐trial interval (ITI) of 6000–8000 ms, during which a white fixation dot (identical to the baseline) was presented. Participants were instructed to observe the cue (S1) and the subsequent emotional image (S2), with no motor response required.

The S2 stimuli consisted of 42 emotional images per database (IAPS, OASIS), each presented twice. The selection included 14 pleasant images (i.e., erotica, sports), 14 neutral images (i.e., neutral scenes, objects), and 14 unpleasant images (i.e., threat, explosions, animals) from each database (IAPS, OASIS), resulting in 28 trials per emotional category and a total of 84 trials per database. To ensure comparable emotional responses elicited by stimuli across databases, images with similar content were selected. Table S1 reports the identification codes and semantic categories of all selected images (see Table S1, Supplementary). A chi‐square test indicated no significant differences in the distribution of semantic categories between the IAPS and OASIS databases (*χ* ^2^_(6)_ = 0.84, *p* = 0.99). Moreover, as the original resolution of OASIS images is lower than that of IAPS images, OASIS stimuli were upscaled using an online tool to approximate the visual properties of the two sets. As a result, the stimuli were rendered nearly equivalent in terms of visual properties (OASIS: 1000 × 800 pixel; IAPS: 1024 × 768 pixel). In addition, mean normative arousal and valence ratings were also matched for each emotional category. Because the IAPS and OASIS databases use different rating scales for valence and arousal (IAPS: 1–9, Lang and Bradley 2007; OASIS: 1–7, Kurdi et al. 2017), ratings were mean‐centered for paired‐sample *t*‐tests comparing normative ratings from the same emotional category between databases (Table 1). Results confirmed that normative valence and arousal ratings were comparable between databases for each emotional category (all *p*s > 0.61). Within both the IAPS and OASIS databases, pleasant pictures had significantly higher normative valence ratings than neutral and unpleasant ones, while neutral pictures had higher ratings than unpleasant ones (all *p*s < 0.001). Moreover, pleasant and unpleasant pictures were matched in arousal levels (all *p*s > 0.74), which were significantly higher than those of neutral pictures (all *p*s < 0.001). To induce greater psychophysiological changes, only highly arousing pleasant and unpleasant pictures were selected (Bradley et al. 2001).

**TABLE 1:** Normative valence and arousal ratings (mean‐centered scores) of the images selected from the IAPS and the OASIS databases.

Normative ratings	Pleasant	Neutral	Unpleasant
IAPS database			
Valence	1.13 ± 0.29	−0.01 ± 0.13	−1.20 ± 0.35
Arousal	0.73 ± 0.17	−1.32 ± 0.34	0.62 ± 0.27
OASIS database			
Valence	1.18 ± 0.21	−0.02 ± 0.24	−1.16 ± 0.27
Arousal	0.75 ± 0.33	−1.35 ± 0.19	0.59 ± 0.19

*Note:* Data are M(SD). IAPS ratings were evaluated on a 1–9 scale, while OASIS ratings were evaluated on a 1–7 scale.

Stimuli were presented in a semi‐randomized sequence to prevent consecutive S1‐S2 pairs from having the same emotional valence. At the end of each block, the 42 images (14 for each emotional category) shown during the S1‐S2 paradigm were presented again, and ratings of emotional valence and arousal were obtained using a computerized version of the 9‐point Valence and Arousal scales of the Self‐Assessment Manikin (SAM; Bradley and Lang 1994).

### 2.3 Procedure

The study was advertised through various channels, including flyers posted around the campus and announcements on online platforms used by University of Padua students, such as Facebook and Telegram groups. Individuals interested in taking part in the study completed an online form via Google Forms, which included an anamnestic interview to assess eligibility and the BDI‐II to assess the presence of depressive symptoms within the preceding 2 weeks. Eligible participants were invited to a laboratory session in the Clinical Psychophysiology laboratory at the Department of General Psychology of the University of Padua. Participants were instructed to refrain from alcohol consumption on the day before the experimental session and to avoid caffeine and nicotine for at least 4 h before the session. Upon arrival at the laboratory, participants read and signed the informed consent form. Then, participants were seated on a comfortable chair in a dimly lit, sound‐attenuated room. This study was part of a broader project that also included the recording of the electrocardiogram (ECG), in addition to the EEG and the administration of a structured interview exploring the family history for psychopathology (Family History Screen, Weissman et al. 2000). After electrode attachment and a 3‐min resting‐state recording, they were shown three practice trials, one for each emotional category (pleasant, neutral, unpleasant). They then took a brief break before completing the second block of the S1‐S2 paradigm with images from the second database, again preceded by three practice trials. The entire procedure took approximately 120 min.

### 2.4 Electroencephalogram Data Acquisition and Analysis

EEG was recorded using a 32‐channel ANT system and a computer running eego software (ANT Neuro, Enschede, Netherlands). The elastic cap with 32 tin electrodes was arranged according to the 10–20 System (Fp1, Fpz, Fp2, F7, F3, Fz, F4, F8, FC5, FC1, FC2, FC6, T7, C3, Cz, C4, T8, CP5, CP1, CP2, CP6, P7, P3, Pz, P4, P8, POz, O1, Oz, O2, and M1 and M2 [mastoids]), with CPz as the online reference. Vertical and horizontal electrooculograms (EOGs) were recorded using a bipolar montage to track horizontal eye movements and blinks, with electrodes placed above and below the right orbit and at the external canthi of the eyes. Electrode impedance was kept below 10 kΩ. The EEG and the EOG signals were recorded in DC with a low‐pass filter of 30 Hz and sampled at 1000 Hz. EEG data was downsampled to 500 Hz and re‐referenced offline to a linked mastoids montage in EEGLAB (Delorme and Makeig 2004). Further analyses were performed in Brainstorm (Tadel et al. 2011). Data was band‐pass filtered from 0.01 to 30 Hz and corrected for blink artifacts using Independent Component Analysis (ICA). Epoching was performed in line with other studies using similar paradigms (e.g., Novak and Foti 2015; Poli et al. 2007). For the Cue‐P300, the EEG signal was segmented into 2300 ms epochs ranging from 300 ms before to 2000 ms after S1 onset; for the SPN, the signal was segmented into 4200 ms epochs, from 2000 to 6200 ms after S1 onset. Finally, being the LPP locked to the emotional stimulus, the EEG signal was segmented to the emotional image (S2) into 2800 ms epochs, spanning from 300 ms before to 2500 ms after its onset. Then, the EEG epochs were semi‐automatically screened for artifacts, defined as amplitude variations exceeding ±100 μV (peak‐to‐peak) for each channel, and contaminated trials were excluded. The remaining epochs were visually inspected to detect and reject any remaining artifacts (e.g., eye movements, muscle activity, segments showing fluctuations greater than ±100 μV). Finally, ERPs were averaged separately for each emotional condition (pleasant, neutral, unpleasant) and baseline‐corrected using the mean activity within the −250 to −50 ms interval preceding the stimulus of interest (S1 for the Cue‐P300 and SPN, S2 for the LPP). Tables S2 and S3 report the average number of trials included for emotional category (pleasant, neutral, unpleasant) and database (IAPS, OASIS), along with the individual standardized measurement errors for each ERP component (see Tables S2 and S3). A repeated measures ANOVA showed no significant differences in the number of accepted trials across emotional categories or databases (all *ps* > 0.34).

The ERP time windows and electrodes were selected based on topographical visualization of the grand average of the three emotional categories and in accordance with previous studies. Specifically, ERP waveforms for the three emotional categories were examined for both databases (IAPS, OASIS). In line with previous studies, the Cue‐P300 was maximal at parietal sites [P3, PZ, P4, P7, P8], while the LPP was maximal at centroparietal [CP1, CP2, CP5, CP6] and parietal [P3, PZ, P4, P7, P8] (e.g., Hajcak and Foti 2020; Moretta et al. 2021). Therefore, the Cue‐P300 was calculated as the averaged peak amplitude^1^ at parietal sites between 200 and 400 ms following S1 onset, while the LPP was scored as the mean amplitude at centroparietal sites within the 300–1000 ms time window following S2 onset. In agreement with previous research (e.g., Buodo et al. 2012; Poli et al. 2007), the SPN was scored as the mean amplitude in the 200 ms preceding the image onset at frontocentral [FC1, FC2, FC5, FC6] and frontal [F3, FZ, F4] sites.

### 2.5 Statistical Analyses

The statistical analyses were performed in Rstudio (R Core Team 2023), particularly the *lme4* (Bates et al. 2014) and *lmerTest* (Kuznetsova et al. 2015) packages. A *p*‐value of 0.05 was the cut‐off value for statistical significance.

To test whether the IAPS and OASIS databases elicited different patterns of self‐reported ratings (valence, arousal) and ERP amplitudes (Cue‐P300, SPN, LPP), different repeated measures linear mixed‐effects models were conducted. All models included the participant as a random intercept, while the Category (pleasant, neutral, unpleasant) and Database (IAPS, OASIS), and their interaction were specified as fixed factors *Model* ← *lmer* (*SAM ratings or ERPs amplitude* ∼ *Category* × *Database* + (1|*Subject*)). *P*‐values were calculated through the Satterthwaite approximation, as implemented in the *lmerTest* package (Kuznetsova et al. 2015). To assess multicollinearity, Variance Inflation Factors (VIF) were examined for all the predictors of the model with the *vif* function of the car package (Fox et al. 2019). Significant categorical effects (*p* < 0.05) were further analyzed by Tukey HSD post‐hoc tests to correct for multiple comparisons.

## 3 Results

### 3.1 Valence and Arousal Self‐Report Ratings

**FIGURE 1:**
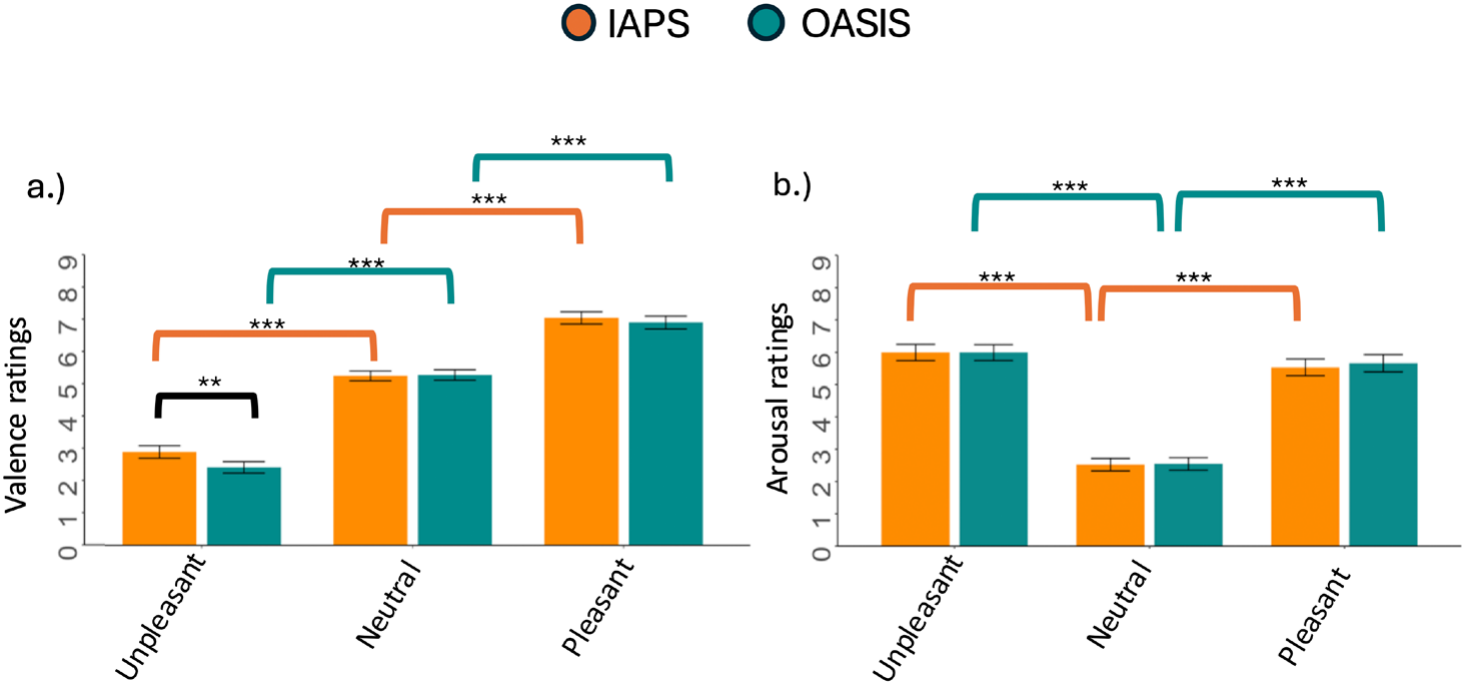
Mean valence (Panel a) and arousal (Panel b) subjective ratings across different emotional categories (unpleasant, neutral, and pleasant) for each database (IAPS, OASIS). Error bars represent the standard error of the mean. Colored square brackets indicate statistically significant contrasts: Orange brackets denote comparisons between emotional categories within the IAPS database, green brackets indicate within‐database comparisons for OASIS, and black brackets represent between‐database comparisons. Asterisks indicate the level of statistical significance (*p < 0.05, **p < 0.01, ***p < 0.001).

The results of the mixed‐effect models on valence ratings showed a significant effect of Category (F_(2, 1696)_ = 1195.39, *p* < 0.0001), Database (F_(1, 1710)_ = 5.95, *p* = 0.01) and Category × Database (F_(2, 1696)_ = 4.18, *p* = 0.02, Figure 1a). Within both databases, valence was higher for pleasant vs. neutral and unpleasant pictures, and higher for neutral vs. unpleasant ones (all *ps*_Tukey_ < 0.001). Moreover, unpleasant pictures within the IAPS database had higher valence ratings than those within the OASIS database (*p*_*Tukey*_ = 0.004).

The results of the mixed‐effect models on arousal rating showed a significant effect of Category (F_(2, 1696)_ = 724.77, *p* < 0.0001, Figure 1b). Specifically, arousal ratings were higher for both pleasant and unpleasant pictures than neutral ones (all *p*s < 0.001). No effect of Database or Database × Category emerged. For both valence and arousal models, VIF values were below 1.72, indicating no concerns regarding multicollinearity, as all values remained under the recommended threshold (Fox et al. 2019).

### 3.2 IAPS vs. OASIS During the Distinct Phases of Emotional Pictures Processing

Table 2 shows the results of the ANOVA performed on the mixed‐effects model using the *lmerTest* (Kuznetsova et al. 2015), which assessed the effects of Category, Database, and their interaction on the amplitude of the three ERPs (Cue‐P300, SPN, LPP). Adjusted VIF values were all below 1.69, indicating no concerns regarding multicollinearity, as all values remained under the recommended threshold of 2.5 (Fox et al. 2019).

**TABLE 2:** Report of the ANOVA performed on three linear mixed‐effects models predicting ERP amplitudes based on Category, Database, and their interaction.

	Sum of squares	df	Mean squares	*F*	*p*
Cue‐P300 model					
Category	**157.95**	**2**	**78.97**	**8.26**	< 0.001***
Database	**256.16**	**1**	**256.16**	**26.81**	< 0.001***
Category × Database	**100.79**	**2**	**50.39**	**5.27**	0.005**
SPN model					
Category	11.59	2	5.79	0.22	0.79
Database	17.75	1	17.75	0.69	0.40
Category × Database	48.89	2	24.44	6.72	0.38
LPP model					
Category	**2806.87**	**2**	**1403.43**	**124.40**	< 0.01**
Database	2.25	1	2.25	0.19	0.65
Category × Database	**267.77**	**2**	**133.88**	**11.86**	< 0.01**

*Note:* The table presents sum of squares, degrees of freedom (df), mean squares, *F*‐values, and *p*‐values for each predictor. Significant results are highlighted in bold, with asterisks indicating the level of statistical significance (*p < 0.05, **p < 0.01, ***p < 0.001).

### 3.3 SPN Results

**FIGURE 2:**
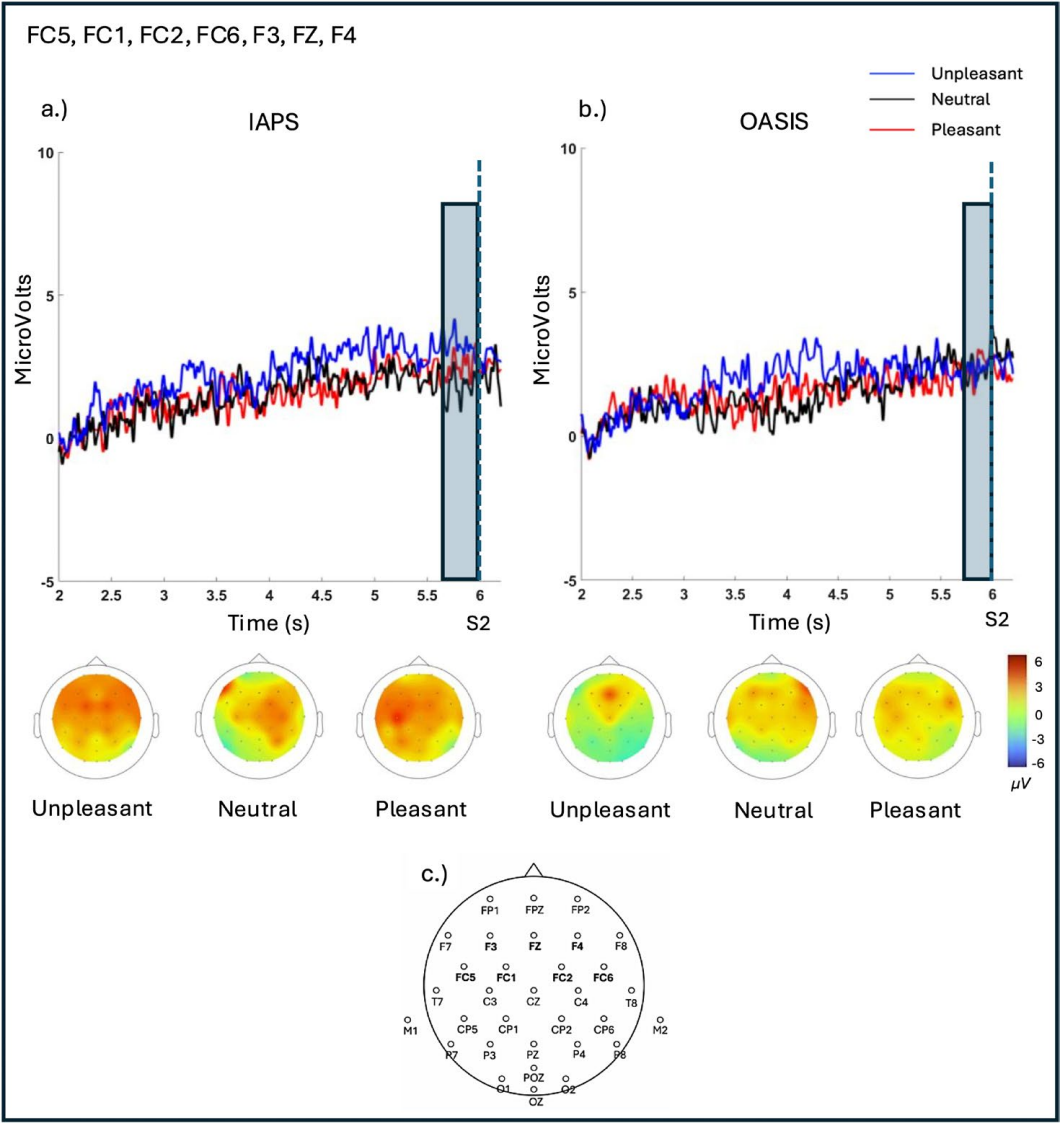
(Panel a) Grand‐average SPN waveforms for IAPS and OASIS (Panel b) images at frontal [F3, FZ, F4] and frontocentral [FC1, FC2, FC5, FC6] electrodes. SPN was scored as the mean amplitude in the shaded window (5.8–6 s). The picture was presented at 6 s. (Panel c) Schematic representation of the 32‐channel EEG montage, indicating the electrodes used for the SPN averaging.

The results of the linear mixed‐effect model predicting the SPN mean amplitude at frontocentral [FC1, FC2, FC5, FC6] and frontal [F3, FZ, F4] electrodes did not yield a significant effect of Category, Database, or Category × Database (all *ps* > 0.38). Consistently, the visual inspection of the grand averages revealed that the SPN did not present the expected pattern of larger negativity to emotional vs. neutral stimuli. Figure 2 shows the grand average SPN at frontocentral [FC1, FC2, FC5, FC6] and frontal [F3, FZ, F4] electrodes in the two databases (IAPS, OASIS) for the three emotional categories (unpleasant, neutral, pleasant), along with the corresponding heatmaps and mean SPN amplitudes.

### 3.4 Emotional Anticipation: Cue‐P300 Elicited by IAPS vs. OASIS Images

**FIGURE 3:**
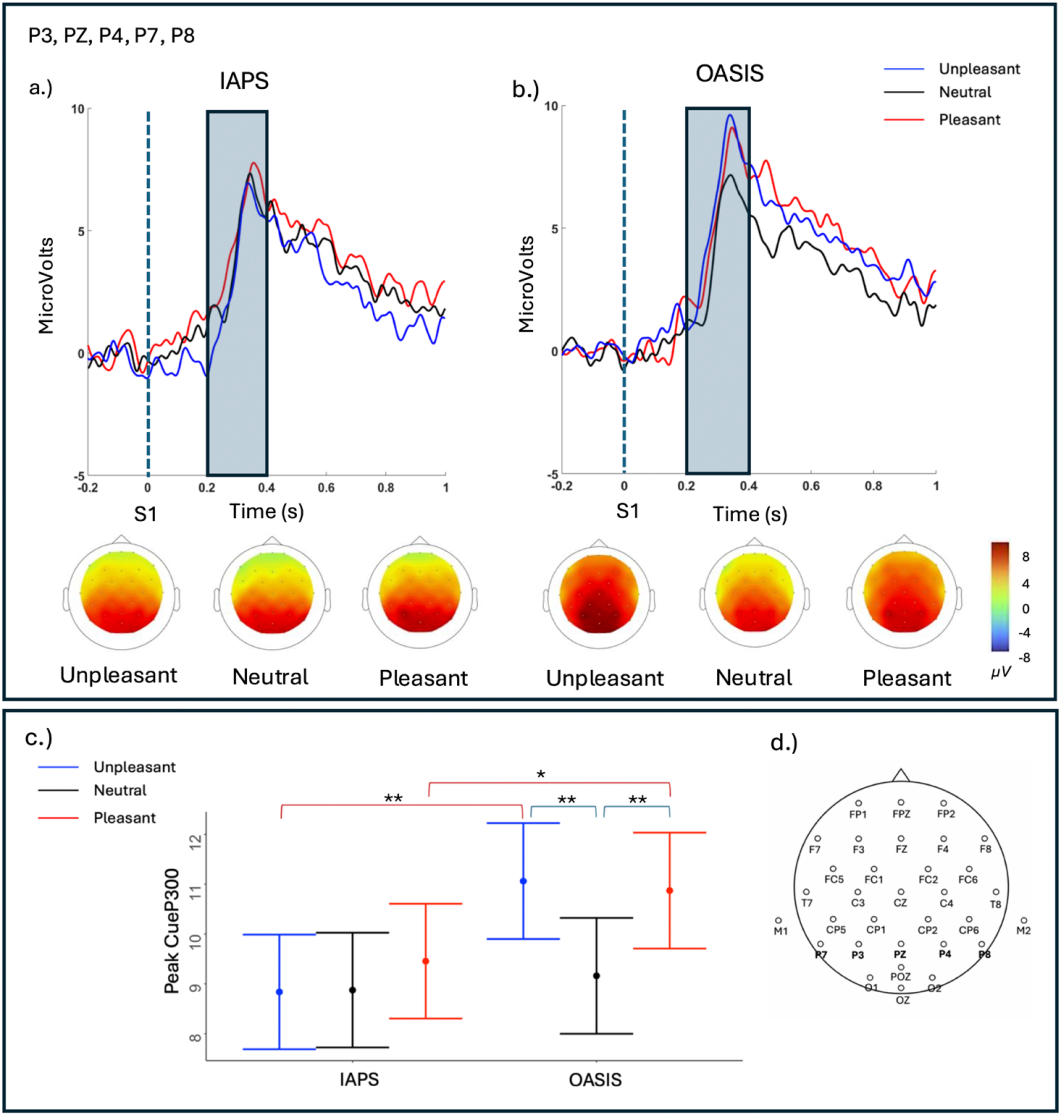
(Panel a) Grand‐average Cue‐P300 waveforms and corresponding heatmaps for IAPS and OASIS (Panel b) images at parietal [P3, PZ, P4, P7, P8] electrodes. Cue‐P300 was scored as the peak amplitude in the shaded window (0.2–0.4 s). The cue (S1) was presented at 0 s. (Panel c) Model predicted mean Cue‐P300 amplitudes for each picture set (IAPS, OASIS) and emotional categories (unpleasant, neutral, pleasant) in the two databases (IAPS, OASIS). Error bars indicate the standard error of the mean. Colored square brackets indicate statistically significant pairwise comparisons: Blue brackets highlight comparisons between emotional categories within the same database, while red brackets indicate comparisons between the two databases for the same emotional category. Asterisks denote significance levels (*p < 0.05, **p < 0.01, ***p < 0.001). (Panel d) Schematic representation of the 32‐channel EEG montage, indicating the electrodes used for the Cue‐P300 averaging.

The results from the linear mixed‐effects model predicting the peak Cue‐P300 amplitude at parietal electrodes showed a significant effect of Category (F_(2, 611.21)_ = 8.27, *p* < 0.001), Database (F_(1, 618.90)_ = 26.81, *p* < 0.001), and Category × Database (F_(2, 611.0.21)_ = 5.27, *p* = < 0.001). Within the IAPS database, no significant difference in Cue‐P300 amplitude emerged across emotional categories (all *ps*_*Tukey*_ > 0.67). In contrast, within the OASIS database, emotional stimuli elicited a larger Cue‐P300 than neutral stimuli (unpleasant vs. neutral, *p*_*Tukey*_ < 0.01, pleasant vs. neutral, *p*_*Tukey*_ < 0.01), while no difference emerged from pleasant and unpleasant stimuli (*p*_*Tukey*_ = 0.99). In addition, emotional images (pleasant, unpleasant) from the OASIS database elicited a larger Cue‐P300 compared to the corresponding IAPS images of the same emotional category (IAPS‐pleasant vs. OASIS‐pleasant, *p*_*Tukey*_ = 0.01; IAPS‐unpleasant vs. OASIS‐unpleasant, *p*_*Tukey*_ < 0.01), while no difference emerged between neutral images between IAPS and OASIS databases (*p*_*Tukey*_ = 0.98). Figure 3 shows the grand average Cue‐P300 at parietal [P3, PZ, P4, P7, P8] electrodes in the two databases (IAPS, OASIS) for the three emotional categories (unpleasant, neutral, pleasant), along with the corresponding heatmaps and mean Cue‐P300 amplitudes.

### 3.5 Emotional Elaboration: LPP Elicited by IAPS vs. OASIS Images

**FIGURE 4:**
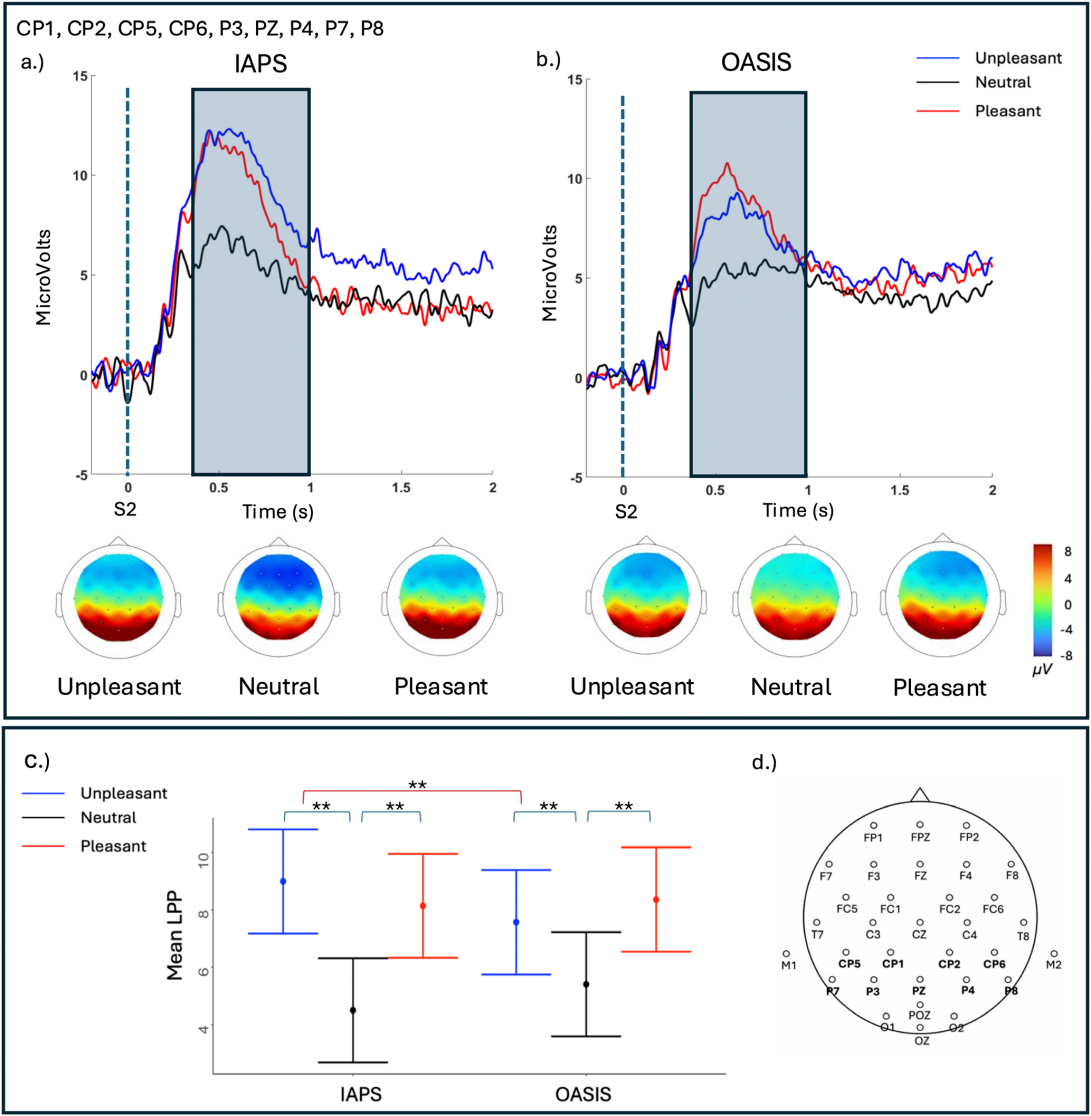
(Panel a) Grand‐average LPP waveforms and corresponding heatmaps for IAPS and OASIS (Panel b) images at centroparietal [CP1, CP2, CP5, CP6] and parietal [P3, PZ, P4, P7, P8] electrodes. LPP was scored as the mean amplitude in the shaded window (6.5–7 s), and the picture was presented at 6 s. (Panel c) Model predicted mean LPP amplitudes for each picture set (IAPS, OASIS) and emotional categories (unpleasant, neutral, pleasant) in the two databases (IAPS, OASIS). Error bars indicate the standard error of the mean. Asterisks highlight significant pairwise comparisons between emotional categories. Colored square brackets indicate statistically significant pairwise comparisons: Blue brackets highlight comparisons between emotional categories within the same database, while red brackets indicate comparisons between the two databases for the same emotional category. Asterisks denote significance levels (*p < 0.05, **p < 0.01, ***p < 0.001). (Panel d) Schematic representation of the 32‐channel EEG montage, indicating the electrodes used for the LPP averaging.

The results from the linear mixed‐effect model predicting LPP amplitude at centro‐parietal sites revealed a significant effect of Category (F_(2, 1123)_ = 124.40, *p* < 0.01), and Category × Database (F_(2, 1123)_ = 11.86, *p* < 0.01). Within both the IAPS and the OASIS databases, pleasant and unpleasant images elicited a larger amplitude than neutral images (pleasant vs. neutral, all *ps*_*Tukey*_ < 0.01, unpleasant vs. neutral, all *ps*_*Tukey*_ < 0.01), whereas no difference between pleasant and unpleasant pictures emerged (all *ps*_*Tukey*_ > 0.12). Moreover, IAPS unpleasant stimuli elicited a larger LPP than OASIS unpleasant stimuli (*p*_*Tukey*_ < 0.01). Figure 4 shows the grand average of the LPP at centroparietal [CP1, CP2, CP5, CP6] and parietal [P3, PZ, P4, P7, P8] electrodes in the two databases (IAPS, OASIS) for the three emotional categories (unpleasant, neutral, pleasant), along with the corresponding heatmaps and mean LPP amplitudes.

## 4 Discussion

While the IAPS remains the most widely used emotional image database in affective psychophysiology, its limitations—such as cultural biases and within‐individual habituation in longitudinal studies—have led to the development of alternative databases, including the OASIS (Kurdi et al. 2017). The present study aimed to compare neural responses (Cue‐P300, SPN, LPP amplitudes) and subjective ratings elicited by IAPS and OASIS emotional images, matched for normative valence and arousal ratings, in a sample of 23 young adults. The primary objective was to assess whether OASIS images could be integrated in psychophysiological experiments, alongside IAPS images, when assessing distinct emotional processing stages. While the two databases elicited distinct patterns of neural responses during emotional anticipation, they both reliably capture core aspects of emotional elaboration, supporting their complementary use.

Regarding the component reflecting affective anticipation, the Cue‐P300 was larger for emotional compared to neutral OASIS images, while no emotional modulation elicited from IAPS images was present for this component. This pattern indicates that, during the anticipatory stage, OASIS images may be more effective in recruiting attentional and preparatory mechanisms in response to emotional cues, possibly due to their higher ecological validity and temporal relevance, which may enhance motivated attention toward forthcoming emotional stimuli. Notably, the Cue‐P300 has been predominantly examined in reward processing paradigms, in which the anticipated stimulus typically consisted of feedback signaling gains or losses (e.g., Novak and Foti 2015; Thompson et al. 2023). Therefore, future studies should systematically examine characteristics of upcoming emotional pictures—such as stimulus novelty or complexity—to elucidate the factors driving Cue‐P300 modulation in response to emotionally salient cues. As for the SPN, this component did not show the expected pattern of larger amplitudes during the anticipation of emotional compared to neutral pictures for both IAPS and OASIS images. This absence of modulation may be due to the paradigm not eliciting sufficient anticipatory mechanisms. One possible explanation is that, to maintain consistency with previous studies, each cue (S1) was congruent with the upcoming emotional image (S2) (Buodo et al. 2012; Poli et al. 2007). As observed in other recent studies, the predictability of this association may have reduced the need to engage anticipatory resources (Dell’Acqua et al. 2025). Hence, further research is needed to systematically manipulate the predictability of the upcoming stimulus to clarify the conditions under which the SPN is modulated. Moreover, the structure of the task—comprising two S1–S2 blocks with images from different databases—might have further reduced the likelihood of detecting an anticipatory component, as participants may have clearly understood the task structure and focused primarily on the content of the images rather than their anticipation.

Moreover, an affective modulation of the LPP was observed for both IAPS and OASIS, with pleasant and unpleasant stimuli eliciting greater LPP amplitudes than neutral ones in both image sets. Notably, LPP was larger to IAPS than OASIS unpleasant images. Because images were matched on semantic content across databases, the effect is unlikely to reflect differences in the type of depicted scenes. Rather, it may reflect disparities in higher‐order emotional content or in the affective salience between OASIS and IAPS unpleasant images. Hence, these findings suggest that both databases effectively elicit emotional processing, but IAPS unpleasant images may convey a greater motivational relevance than their OASIS counterparts. However, even if previous research has demonstrated that the LPP is largely independent of low‐level perceptual properties, such as picture size, complexity, and color (Ferrari et al. 2017), subtle and less easily controllable perceptual differences cannot be entirely ruled out. Future studies are warranted to systematically control for perceptual properties while comparing LPP amplitude in response to emotional stimuli of different databases.

At the subjective level, as expected, valence was higher for pleasant relative to neutral and unpleasant pictures, and higher for neutral relative to unpleasant ones across both the IAPS and OASIS databases. However, unpleasant pictures were rated as more unpleasant in the OASIS database relative to the IAPS database. Arousal ratings followed the anticipated pattern, with both pleasant and unpleasant images eliciting higher arousal than neutral images, and no significant differences in arousal ratings emerged between the two databases. Overall, these findings suggest that the IAPS and OASIS databases are largely comparable in terms of subjective valence and arousal ratings.

Taken together, these results indicate that both IAPS and OASIS images reliably elicit the expected emotional responses. Self‐reported ratings were similar across the two databases, together with the well‐established modulation of the LPP by emotional content. Regarding the ERPs reflecting emotional anticipation, differences in the Cue‐P300 amplitude were observed, with emotional modulation emerging for OASIS but not IAPS images. However, these effects should be interpreted with caution, as they may reflect idiosyncratic variations rather than systematic differences between databases. Furthermore, the absence of a detectable SPN suggests that the present paradigm was not optimal for capturing emotional anticipation. Future research employing paradigms that more effectively elicit this component could help clarify whether IAPS and OASIS sets are comparable, or might differ, at this stage. Furthermore, the LPP results show that IAPS images continue to perform as expected, eliciting a robust emotional modulation despite concerns about their dated content. This provides compelling rationale for researchers to continue relying on this widely used dataset. Nonetheless, integrating IAPS with alternative sets could still be advantageous, enhancing the diversity and ecological validity of the stimuli presented in emotional processing paradigms. For instance, future studies should explore the efficacy of OASIS images in eliciting ERP responses in comparison with other databases, such as emoMadrid (Carretié et al. 2019), to determine their relative effectiveness in capturing different stages of emotional processing. Additionally, a multimodal approach integrating multiple psychophysiological measures (e.g., skin conductance) could provide further insights into the specific attributes of affective images that drive psychophysiological responses.

This study has several limitations that should be acknowledged. First, the sample was relatively small and consisted exclusively of young university students, which limits the generalizability of the findings. Future research with a larger and more diverse sample could enhance the robustness and applicability of these conclusions. Nevertheless, the affective modulation of the LPP in response to IAPS images was consistently observed within this group, supporting the reliability of the findings in this context. Second, although individuals with current depressive symptoms were excluded, other factors may have influenced the modulation of the ERPs amplitudes, such as anxiety symptoms (Weinberg et al. 2016), personality features (Speed et al. 2015), or a parental history of depression (e.g., Moretta and Messerotti Benvenuti 2023). Third, this study relied on a limited subset of images from each database, which may constrain the generalizability of results to the full image sets. However, to maximize the likelihood of affective modulation, images with the highest arousal ratings were specifically selected for both pleasant and unpleasant categories.

In summary, the present study provides the first direct comparison of ERP components reflecting emotional anticipation (Cue‐P300, SPN) and emotional elaboration (LPP) elicited by images from the IAPS and OASIS databases. The findings indicate that both databases successfully elicited robust LPP modulation, with IAPS images generating stronger emotional responses to unpleasant stimuli. In contrast, OASIS images evoked larger Cue‐P300 amplitudes, particularly for unpleasant stimuli, suggesting that specific features of this database may enhance anticipatory processing. These results offer valuable methodological implications for affective psychophysiology research, emphasizing the importance of stimulus selection in shaping emotional and neural responses.

## Author Contributions

**Valentina Mologni:** conceptualization, investigation, writing – original draft, methodology, visualization, formal analysis, data curation. **Carola Dell'Acqua:** conceptualization, investigation, funding acquisition, writing – review and editing, methodology, supervision, data curation. **Simone Messerotti Benvenuti:** conceptualization, investigation, funding acquisition, methodology, writing – review and editing, supervision.

## Funding

We acknowledge financial support under the National Recovery and Resilience Plan (NRRP) funded by the European Union—NextGenerationEU—Project Numbers: 20228P4H2K and P20223PTH4, adopted by the Italian Ministry of University and Research (MUR).

## Conflicts of Interest

The authors declare no conflicts of interest.

## Supporting information


**Table S1:** Identification codes of IAPS and OASIS images listed by semantic category.
**Table S2:** Standardized measurement errors (SME) and number of trials (*n*) included for each condition (pleasant, neutral, unpleasant) for ERP (Cue‐P300, SPN, LPP) in the IAPS database. Also reported are the root mean square of the standardized measurement error values (RMS(SME)), and the average number of included trials.
**Table S3:** Standardized measurement errors (SME) and number of trials (*n*) included for each condition (pleasant, neutral, unpleasant) for ERP (Cue‐P300, SPN, LPP) in the OASIS database. Also reported are the root mean square of the standardized measurement error values (RMS(SME)), and the average number of included trials.

## Data Availability

The data that support the findings of this study are available from the corresponding author upon reasonable request.

## Endnotes

^1^
For the Cue‐P300, mean amplitude data were analyzed in the same temporal window, yielding results consistent with the peak amplitude analyses.
