# Supplementary material for: Replacing the Classics? A Comparison of the ERPs Evoked by IAPS and OASIS Images During Emotional Processing
Source: Psychophysiology. 2026 Jan 20;63(1):e70231. doi: 10.1111/psyp.70231 (PMC12819365; doi:10.1111/psyp.70231)
Supplement: Supplementary file 1 — Table S1: Identification codes of IAPS and OASIS images listed by semantic category. Table S2: Standardized measurement errors (SME) and number of trials (n) included for each condition (pleasant, neutral, unpleasant) for ERP (Cue‐P300, SPN, LPP) in the IAPS database. Also reported are the root mean square of the standardized measurement error values (RMS(SME)), and the average number of included trials. Table S3: Standardized measurement errors (SME) and number of trials (n) included for each condition (pleasant, neutral, unpleasant) for ERP (Cue‐P300, SPN, LPP) in the OASIS database. Also reported are the root mean square of the standardized measurement error values (RMS(SME)), and the average number of included trials. [file PSYP-63-e70231-s001.docx]

**Replacing the classics? A comparison of the ERPs evoked by IAPS and OASIS images during emotional processing**

*Mologni et al*.

**Table S1.** Identification codes of IAPS and OASIS images listed by semantic category.

| **Semantic Category** | **IAPS** | **OASIS** |
| --- | --- | --- |
| **Pleasant - Sports** | 5626, 8034, 8080, 8180, 8185, 8200, 8300, 8370, 8400, 8490 | Bunjee jumping 2, Cliff diver 3, Parachuting 3, Rafting 2, Rafting 5, Rollercoaster 1, Sailing 1, Skydiving 1 |
| **Pleasant - Erotica** | 4611, 4651, 4652, 4695 | Nude couple 5, Nude couple 6, Nude couple 8, Nude couple 11, Nude couple 12, Nude couple 14 |
| **Neutral – Objects** | 5332, 7002, 7004, 7009, 7036, 7041, 7224, 7547 | Bottle 1, Fence 1, Fence 5, Roofing 4, Snow 4, Storage 3, Timber 1, Windmill 1 |
| **Neutral – Scenes** | 2493, 2495, 2512, 2575, 2595, 2840 | Bored pose 1, Bored pose 2, Bored pose 3, Neutral pose 3, Sleepy pose 2, Sleepy pose 3, |
| **Unpleasant – Threat** | 6200, 6250, 6260, 6312 | Car crash 1, Car crash 3, War 1, War 2, War 6, |
| **Unpleasant - Explosions** | 9622, 9630, 9635.2, 9909, 9940 | Explosion 2, Explosion 6, Fire 5, Fire 7 |
| **Unpleasant – Animals** | 1120, 1201, 1301, 1525, 9185 | Animal carcass 5, Animal carcass 6, Dog 24, Dog 26, Snake 4 |

*Notes:* To ensure comparable image resolution across the two databases, OASIS images were upscaled using an online image resizing tool (*https://www.iloveimg.com*).

**Table S2.** Standardized measurement errors (SME) and number of trials (*n*) included for each condition (pleasant, neutral, unpleasant) for ERP (Cue-P300, SPN, LPP) in the IAPS database. Also reported are the root mean square of the standardized measurement error values (RMS(SME), and the average number of included trials.

|  | IAPS | | | | | | | | | | | | | | | | | | | | | | | | | | |
| --- | --- | --- | --- | --- | --- | --- | --- | --- | --- | --- | --- | --- | --- | --- | --- | --- | --- | --- | --- | --- | --- | --- | --- | --- | --- | --- | --- |
|  | ***Peak amplitude Cue-P300*** | | | | | | ***Mean amplitude SPN*** | | | | | | | | | ***Mean amplitude LPP*** | | | | | | | | | | | |
|  | **Pleasant** | | **Neutral** | | **Unpleasant** | | **Pleasant** | | **Neutral** | | | | **Unpleasant** | | | **Pleasant** | | | | **Neutral** | | | | **Unpleasant** | | | |
| Subj (ID) | SME | Trials (n) | SME | Trials (n) | SME | Trials (n) | SME | Trials (n) | SME | | Trials (n) | | SME | | Trials (n) | SME | | Trials (n) | | SME | | Trials (n) | | SME | | Trials (n) |  |
| 1 | 1.18 | 28 | 1.13 | 28 | 1.15 | 28 | 2.52 | 28 | 2.03 | 27 | | 2.39 | | 28 | | 1.27 | 28 | | 1.26 | | 28 | | 1.21 | | 28 | |  |
| 2 | 1.47 | 28 | 1.78 | 28 | 1.49 | 28 | 2.46 | 28 | 2.74 | 28 | | 3.11 | | 27 | | 1.98 | 28 | | 1.95 | | 28 | | 1.94 | | 28 | |  |
| 3 | 1.96 | 28 | 1.66 | 28 | 2.15 | 27 | 2.41 | 27 | 2.43 | 28 | | 3.31 | | 27 | | 1.91 | 28 | | 1.60 | | 28 | | 2.24 | | 27 | |  |
| 4 | 1.78 | 28 | 1.66 | 28 | 2.33 | 28 | 6.72 | 28 | 6.72 | 27 | | 4.87 | | 28 | | 1.90 | 28 | | 2.04 | | 28 | | 2.01 | | 28 | |  |
| 5 | 2.78 | 27 | 3.44 | 27 | 1.98 | 26 | 3.46 | 26 | 4.21 | 26 | | 5.02 | | 26 | | 3.03 | 28 | | 2.27 | | 28 | | 2.24 | | 28 | |  |
| 6 | 1.50 | 27 | 1.71 | 28 | 1.36 | 27 | 4.78 | 28 | 5.22 | 28 | | 6.50 | | 27 | | 2.03 | 28 | | 1.97 | | 28 | | 1.58 | | 27 | |  |
| 7 | 2.19 | 27 | 1.41 | 25 | 2.19 | 22 | 6.91 | 24 | 7.81 | 20 | | 9.68 | | 23 | | 2.27 | 25 | | 3.03 | | 27 | | 2.35 | | 24 | |  |
| 8 | 1.63 | 24 | 1.40 | 25 | 2.41 | 24 | 3.55 | 24 | 3.64 | 23 | | 5.25 | | 19 | | 2.08 | 26 | | 1.30 | | 25 | | 1.45 | | 23 | |  |
| 9 | 1.99 | 26 | 1.77 | 23 | 1.76 | 21 | 8.54 | 22 | 7.14 | 21 | | 6.66 | | 23 | | 1.72 | 25 | | 2.07 | | 20 | | 3.01 | | 23 | |  |
| 10 | 1.27 | 28 | 1.34 | 28 | 1.96 | 28 | 1.56 | 28 | 1.16 | 28 | | 1.32 | | 28 | | 0.94 | 28 | | 1.31 | | 28 | | 1.20 | | 28 | |  |
| 11 | 3.10 | 28 | 3.20 | 28 | 3.49 | 27 | 6.75 | 25 | 4.96 | 26 | | 5.83 | | 25 | | 2.59 | 27 | | 4.03 | | 27 | | 2.81 | | 27 | |  |
| 12 | 1.31 | 28 | 1.82 | 28 | 1.22 | 28 | 3.69 | 27 | 3.34 | 28 | | 2.78 | | 26 | | 1.87 | 28 | | 1.41 | | 28 | | 1.60 | | 28 | |  |
| 13 | 1.30 | 28 | 1.81 | 27 | 1.22 | 28 | 2.50 | 27 | 3.00 | 28 | | 3.72 | | 27 | | 1.02 | 28 | | 1.15 | | 26 | | 1.56 | | 26 | |  |
| 14 | 2.00 | 26 | 2.04 | 28 | 2.13 | 27 | 6.15 | 27 | 5.63 | 28 | | 3.85 | | 27 | | 3.44 | 28 | | 2.44 | | 28 | | 2.78 | | 27 | |  |
| 15 | 1.46 | 28 | 1.39 | 28 | 1.27 | 28 | 2.53 | 28 | 2.46 | 28 | | 1.87 | | 28 | | 0.99 | 28 | | 1.60 | | 28 | | 1.35 | | 27 | |  |
| 16 | 1.89 | 27 | 1.74 | 28 | 3.44 | 23 | 5.87 | 22 | 2.95 | 22 | | 6.11 | | 22 | | 1.71 | 25 | | 1.99 | | 24 | | 2.39 | | 27 | |  |
| 17 | 1.93 | 27 | 1.50 | 28 | 2.22 | 28 | 4.92 | 25 | 2.47 | 26 | | 4.61 | | 28 | | 4.02 | 28 | | 1.85 | | 26 | | 1.69 | | 28 | |  |
| 18 | 2.94 | 27 | 2.04 | 28 | 2.23 | 25 | 4.32 | 24 | 3.91 | 26 | | 6.02 | | 25 | | 3.41 | 24 | | 2.17 | | 26 | | 2.05 | | 28 | |  |
| 19 | 2.53 | 28 | 1.94 | 28 | 1.89 | 28 | 5.28 | 27 | 6.37 | 25 | | 4.46 | | 27 | | 2.76 | 27 | | 2.47 | | 27 | | 3.34 | | 27 | |  |
| 20 | 1.77 | 28 | 1.37 | 28 | 1.70 | 28 | 1.16 | 28 | 1.17 | 28 | | 1.68 | | 27 | | 1.03 | 28 | | 1.40 | | 28 | | 1.47 | | 27 | |  |
| 21 | 2.90 | 16 | 1.79 | 15 | 2.24 | 17 | 3.23 | 17 | 3.63 | 14 | | 5.03 | | 17 | | 2.50 | 15 | | 2.47 | | 14 | | 1.83 | | 17 | |  |
| 22 | 1.91 | 27 | 1.15 | 28 | 1.38 | 26 | 4.05 | 27 | 1.70 | 28 | | 2.40 | | 27 | | 1.96 | 28 | | 1.22 | | 28 | | 1.52 | | 27 | |  |
| ***RMS (SME)*** | **2.03** |  | **1.86** |  | **2.06** |  | **4.65** |  | **4.29** |  | | **4.80** | |  | | **2.35** |  | | **2.07** | |  | | **2.07** | |  | |  |
| ***Trials M(SD)*** |  | **26.77 (2.59)** |  | **26.77 (2.94)** |  | **26.36 (2.59)** |  | **25.77 (2.75)** |  | **25.59 (3.58)** | |  | | **25.54 (3.00)** | |  | **26.64 (2.90)** | |  | | **26.27 (3.34)** | |  | | **26.36 (2.59)** | |  |

**Table S3.** Standardized measurement errors (SME) and number of trials (*n*) included for each condition (pleasant, neutral, unpleasant) for ERP (Cue-P300, SPN, LPP) in the OASIS database. Also reported are the root mean square of the standardized measurement error values (RMS(SME), and the average number of included trials.

|  | OASIS | | | | | | | | | | | | | | | | | | | | | | | | | | | | | | | |
| --- | --- | --- | --- | --- | --- | --- | --- | --- | --- | --- | --- | --- | --- | --- | --- | --- | --- | --- | --- | --- | --- | --- | --- | --- | --- | --- | --- | --- | --- | --- | --- | --- |
|  | ***Peak amplitude Cue-P300*** | | | | | | | ***Mean amplitude SPN*** | | | | | | | | | | | | ***Mean amplitude LPP*** | | | | | | | | | | | | |
|  | **Pleasant** | | **Neutral** | | **Unpleasant** | | | **Pleasant** | | | **Neutral** | | | | | **Unpleasant** | | | | **Pleasant** | | | | | **Neutral** | | | | | **Unpleasant** | | |
| Subj (ID) | SME | Trials (n) | SME | Trials (n) | SME | Trials (n) | SME | | Trials (n) | SME | | | Trials (n) | | SME | | | Trials (n) | SME | | Trials (n) | | | SME | | | Trials (n) | | SME | | | Trials (n) |
| 1 | 1.23 | 28 | 1.52 | 28 | 1.39 | 28 | 2.71 | | 28 | 2.08 | | 28 | | 2.36 | | | 28 | | 1.55 | | | 28 | 1.65 | | | 28 | | 1.35 | | | 28 | |
| 2 | 0.92 | 28 | 0.92 | 28 | 0.97 | 26 | 2.72 | | 28 | 2.81 | | 27 | | 3.36 | | | 27 | | 1.37 | | | 26 | 1.11 | | | 28 | | 0.79 | | | 28 | |
| 3 | 1.56 | 28 | 1.67 | 27 | 1.92 | 28 | 3.24 | | 27 | 3.12 | | 26 | | 3.87 | | | 28 | | 1.83 | | | 26 | 2.07 | | | 27 | | 1.38 | | | 27 | |
| 4 | 1.63 | 28 | 1.56 | 28 | 2.08 | 28 | 3.84 | | 25 | 4.11 | | 28 | | 3.84 | | | 27 | | 1.77 | | | 28 | 1.82 | | | 28 | | 1.97 | | | 27 | |
| 5 | 1.91 | 27 | 1.93 | 28 | 2.65 | 27 | 6.50 | | 25 | 5.67 | | 26 | | 5.02 | | | 27 | | 2.52 | | | 28 | 1.80 | | | 27 | | 2.93 | | | 26 | |
| 6 | 2.22 | 28 | 1.81 | 28 | 1.89 | 28 | 3.82 | | 27 | 4.83 | | 27 | | 5.24 | | | 27 | | 1.85 | | | 27 | 2.32 | | | 28 | | 1.61 | | | 28 | |
| 7 | 1.54 | 27 | 1.66 | 25 | 1.91 | 27 | 7.03 | | 27 | 7.16 | | 26 | | 7.90 | | | 27 | | 2.36 | | | 26 | 3.38 | | | 25 | | 2.74 | | | 28 | |
| 8 | 2.45 | 22 | 2.01 | 25 | 1.92 | 24 | 5.02 | | 20 | 5.36 | | 24 | | 5.48 | | | 23 | | 2.18 | | | 24 | 2.00 | | | 24 | | 2.27 | | | 22 | |
| 9 | 1.26 | 22 | 2.57 | 22 | 1.83 | 25 | 5.79 | | 22 | 6.06 | | 27 | | 6.89 | | | 22 | | 2.57 | | | 28 | 1.66 | | | 27 | | 2.55 | | | 26 | |
| 10 | 1.38 | 27 | 1.18 | 27 | 0.99 | 28 | 1.67 | | 27 | 1.72 | | 28 | | 1.35 | | | 26 | | 1.42 | | | 28 | 0.96 | | | 27 | | 1.14 | | | 27 | |
| 11 | 5.23 | 23 | 2.70 | 24 | 3.78 | 24 | 5.48 | | 17 | 4.99 | | 25 | | 7.77 | | | 18 | | 2.94 | | | 24 | 3.62 | | | 23 | | 3.88 | | | 27 | |
| 12 | 1.81 | 28 | 1.43 | 28 | 1.32 | 27 | 5.37 | | 28 | 4.40 | | 28 | | 5.48 | | | 27 | | 1.58 | | | 28 | 2.11 | | | 28 | | 1.87 | | | 28 | |
| 13 | 2.55 | 28 | 2.20 | 27 | 2.03 | 28 | 4.51 | | 28 | 6.38 | | 28 | | 3.85 | | | 28 | | 2.55 | | | 28 | 2.04 | | | 28 | | 1.57 | | | 28 | |
| 14 | 2.07 | 26 | 1.67 | 25 | 1.83 | 26 | 3.21 | | 28 | 2.71 | | 25 | | 2.88 | | | 27 | | 1.59 | | | 28 | 1.65 | | | 27 | | 1.53 | | | 28 | |
| 15 | 1.43 | 28 | 1.54 | 28 | 1.75 | 28 | 1.44 | | 28 | 2.00 | | 28 | | 1.86 | | | 28 | | 1.42 | | | 28 | 1.37 | | | 27 | | 1.34 | | | 27 | |
| 16 | 3.88 | 28 | 3.85 | 26 | 3.20 | 22 | 7.58 | | 22 | 6.16 | | 26 | | 8.42 | | | 20 | | 3.52 | | | 24 | 4.01 | | | 24 | | 3.20 | | | 21 | |
| 17 | 1.40 | 25 | 1.50 | 25 | 1.65 | 28 | 3.40 | | 24 | 3.19 | | 24 | | 3.67 | | | 26 | | 2.17 | | | 25 | 2.06 | | | 26 | | 1.77 | | | 26 | |
| 18 | 2.48 | 26 | 1.46 | 28 | 1.70 | 27 | 5.32 | | 24 | 5.52 | | 28 | | 4.56 | | | 27 | | 2.62 | | | 26 | 1.85 | | | 27 | | 2.40 | | | 28 | |
| 19 | 2.06 | 28 | 1.95 | 28 | 2.01 | 28 | 1.41 | | 27 | 1.44 | | 27 | | 1.35 | | | 28 | | 1.33 | | | 26 | 1.23 | | | 28 | | 1.24 | | | 28 | |
| 20 | 2.26 | 27 | 1.48 | 28 | 2.25 | 27 | 5.61 | | 26 | 4.13 | | 27 | | 4.45 | | | 27 | | 1.41 | | | 28 | 1.76 | | | 24 | | 1.35 | | | 27 | |
| 22 | 1.61 | 28 | 1.05 | 28 | 1.28 | 28 | 5.76 | | 28 | 4.63 | | 28 | | 2.52 | | | 28 | | 1.82 | | | 28 | 1.67 | | | 28 | | 1.76 | | | 28 | |
| ***RMS (SME)*** | **2.37** |  | **1.90** |  | **2.03** |  | **4.89** | |  | **4.52** | |  | | **4.84** | | |  | | **2.10** | | |  | **2.15** | | |  | | **2.08** | | |  | |
| ***Trials M(SD)*** |  | **26.66 (2.00)** |  | **26.71 (1.73)** |  | **26.76 (1.70)** |  | | **25.52 (3.06)** |  | | **26.71 (1.34)** | |  | | | **26.00 (2.81)** | |  | | | **26.76 (1.51)** |  | | | **26.61 (1.62)** | |  | | | **26.80 (1.91)** | |
